# Cardiac surgery with concomitant atrial ablation

**DOI:** 10.1186/1749-8090-10-S1-A334

**Published:** 2015-12-16

**Authors:** Torulv Holst, Mehdy RoostaAzad, Hannes Naegele, Hamid Bigdeli, Majd MakariousLaham, Afsaneh Zandi, Markus Kamler

**Affiliations:** 1 Department of Cardiothoracic Surgery, Heartcenter Essen-Huttrop gGmbh, Essen, 45138, Germany

## Background/Introduction

Concomitant surgical ablation is recommended in patients undergoing cardiac surgery with atrial fibrillation (AF) to increase the incidence of sinus rhythm (SR). According to literature Cox IV seems to be superior to other surgical methods, although more invasive.

## Aims/Objectives

The aim of our study was to analyze our institutional results with pulmonary vein isolation (PVI) using a bipolar radiofrequency clamp in combination with occlusion of the left atrial appendage (LAA).

## Method

Retrospectively we studied our institutional database for all PVI cases. Incidence of normal SR versus AF was evaluated beyond 3 month after surgery. Supplementary follow up (FU) involved incidence of stroke and use of anticoagulants, patients NYHA state, survival and reintervention rate. We separated two groups in either preoperative paroxysmal AF (group 1) or chronic AF (group 2).

## Results

Between 01/2013 and 03/2015 56 patients (32% female; 73 ± 7 years) received PVI and LAA occlusion concomitant to other cardiac surgery. 34 (61%) patients suffered for paroxysmal AF and 22 (39%) for chronic AF. 15 patients underwent isolated mitral valve surgery, 8 in combination with CABG and 3 with aortic valve surgery. 6 patients were admitted for isolated aortic valve surgery, 3 in combination with CABG and 21 for isolated CABG. Median follow up was 267 (102; 365) days after surgery, 84% had normal SR (group 1 85%, group 2 82%, p = 0.59) and there were no strokes and no reinterventions. 75% were on treatment with anticoagulants and 54% were in NYHA class 0-1, 38% in NYHA class 2-3 and 7% in NYHA class 4. We observed 3 deaths, including 1 early death that was lost to FU.

## Discussion/Conclusion

Less invasive PVI in combination with LAA occlusion is safe and reveals high success rates. High rate of anticoagulation despite normal SR at FU has to be analyzed further. Patients undergoing cardiac surgery should receive concomitant PVI and LAA occlusion if AF is present.

